# Description and evaluation of an EBM curriculum using a block rotation

**DOI:** 10.1186/1472-6920-4-19

**Published:** 2004-10-11

**Authors:** David H Thom, Julie Haugen, Peter S Sommers, Peter Lovett

**Affiliations:** 1 Department of Family and Community Medicine, University of California, San Francisco, San Francisco General Hospital, 1001 Potrero Avenue, Building 80/83, San Francisco, CA 94110, USA; 2 Barnett-Briggs Medical Library, University of California, San Francisco, San Francisco General Hospital, 1001 Potrero Avenue, Building 30, San Francisco, CA 94110, USA

## Abstract

**Background:**

While previous authors have emphasized the importance of integrating and reinforcing evidence-based medicine (EBM) skills in residency, there are few published examples of such curricula. We designed an EBM curriculum to train family practice interns in essential EBM skills for information mastery using clinical questions generated by the family practice inpatient service. We sought to evaluate the impact of this curriculum on interns, residents, and faculty.

**Methods:**

Interns (n = 13) were asked to self-assess their level of confidence in basic EBM skills before and after their 2-week EBM rotation. Residents (n = 21) and faculty (n = 12) were asked to assess how often the answers provided by the EBM intern to the inpatient service changed medical care. In addition, residents were asked to report how often they used their EBM skills and how often EBM concepts and tools were used in teaching by senior residents and faculty. Faculty were asked if the EBM curriculum had increased their use of EBM in practice and in teaching.

**Results:**

Interns significantly increased their confidence over the course of the rotation. Residents and faculty felt that the answers provided by the EBM intern provided useful information and led to changes in patient care. Faculty reported incorporating EBM into their teaching (92%) and practice (75%). Residents reported applying the EBM skills they learned to patient care (86%) and that these skills were reinforced in the teaching they received outside of the rotation (81%). All residents and 11 of 12 faculty felt that the EBM curriculum had improved patient care.

**Conclusions:**

To our knowledge, this is the first published EBM curriculum using an individual block rotation format. As such, it may provide an alternative model for teaching and incorporating EBM into a residency program.

## Background

Evidence-based medicine (EBM) strives to provide a systematic approach to integrating the best research evidence with clinician expertise and patient preferences to provide better patient care [1]. While the potential for answering clinical questions using online resources is high [2], a recent study found resident physicians rarely used web-based or other evidence-based sources to answer clinical questions, preferring instead another person or a pocket reference [3]. Many authors have argued for the importance of teaching EBM skills during residency training, and several have cited evidence to support the desirability of integrating EBM training with other aspects of the residency program [4-8]. Such a curriculum presents several challenges, including finding sufficient time to teach EBM skills to interns and developing ways to integrate and reinforce EBM among residents and faculty.

While there have been several studies of residency EBM curricula [4,9-11], none, to our knowledge, has operated in the framework of an intern block rotation. In this paper we describe an EBM curriculum based on an individual block rotation and designed to integrate and reinforce EBM skills throughout the residency program; we also report on its evaluation by interns, residents and faculty.

## Methods

### Setting

The UCSF family practice residency program is based at San Francisco General Hospital (SFGH), a large county hospital serving the urban poor. The program runs a busy family practice inpatient service (at SFGH) as well as the Family Health Center, an outpatient clinic that includes both continuity practices and acute care services. Within this setting, we formulated 3 primary goals for our EBM curriculum: (1) to teach interns basic EBM concepts and skills; (2) to disseminate and reinforce EBM skills to second and third year residents and faculty; and (3) to apply EBM to the care of patients. This study was approved by the UCSF Committee on Human Research.

### Curriculum

The EBM curriculum for UCSF family practice residents began, in its current form, summer of 2001. The core of the curriculum is a 2-week, individual EBM rotation for interns. In contrast to the usual format of multiple lectures or learning modules scattered throughout residency, the two-week individual block format provides for a concentrated time in which to learn EBM. It also allows tailoring of the rotation to fit the residents' backgrounds and interests. During each of the two weeks, residents have 3 half-day clinics, with the remainder of the time available for EBM. The EBM portion of the rotation fulfills the ACGME requirements for resident research and scholarly activity.

The rotation begins with a meeting with the EBM faculty Director (DT) during which the structure and goals of the rotation are communicated, the intern's knowledge and experience related to EBM are assessed by review of his or her experience and by a pre-rotation test of EBM skills and knowledge (described below). Interns receive reference materials [1,12] and a binder including detailed instructions for the rotation and key articles. The intern, together with the rotation faculty director (DT) and the medical librarian co-director (JH), attends family practice inpatient service rounds to obtain one or two clinical questions directly bearing on the care of one or two patients. With faculty guidance, questions are then formatted into the standard EBM structure identified by the acronym PICO (for patient/problem, intervention, comparison, and outcome). The entire process generally takes 5 to 10 minutes and includes modeling of how to formulate an appropriate 'answerable' question by senior residents and the inpatient attending faculty. The question(s) generated are then used by the librarian Co-director as material for a tutorial on developing search strategies and using high-quality web-based EBM resources. The emphasis of the tutorial is to introduce the intern to the concept of information mastery [5]. An initial assessment of the intern's searching experiences and use of EBM resources helps to tailor the tutorial session. In the tutorial, the intern is introduced to essential EBM concepts, including clinical question development, levels of evidence search strategies, and appraisal techniques [13], and learns to translate the 'answerable' clinical question into a 'searchable' one. Based on the type of clinical question, the intern reviews the possible levels of evidence and study designs and formulates a valid search strategy. As the intern searches for the evidence, the medical librarian provides input into alternative search strategies and information resources. The results of this and any subsequent searches are discussed with the faculty EBM Director and a 1-page response is developed in the form of a critically appraised topic or CAT [1]. Each CAT is structured to include the 'clinical bottom line' answer to the question; the clinical scenario that generated the questions; a summary of the evidence; a critical appraisal of the evidence; and citations. Results are presented to and discussed with the inpatient team. CATs are stored at the EBM website for future reference by residents and faculty [14].

During the first week, the intern completes a web-based EBM tutorial [15] which covers critical evaluation of articles about diagnosis, therapy, prognosis, meta-analysis and decision making, and how to ask clinical questions. In the second week the intern receives another 1 or 2 clinical questions from the inpatient service which are searched and answered as above. The intern also prepares and presents a journal club, which is attended by faculty, residents and medical students. A research article is selected by the intern (with guidance from the EBM Director), which could potentially change a primary care practice around a clinical question. The article is critically reviewed using an EBM approach [1,12], presented and discussed by the group.

During the initial 3 months of the curriculum, interns completed a written test of their EBM skills and knowledge before and immediately following the rotation. The tests were adapted, with permission, from a similar instrument developed by Sean Schafer, MD and Katie Ramos, PhD at the University of California Fresno Family Practice Residency Program [16]. Scores improved post-rotation in all 3 areas tested: EBM terms and concepts 81% to 97%; quantitative skills 51% to 80%; question formulation and searching 71% to 92%, with the total score increasing from 63% to 87%.

As others have noted, progress in the use of EBM depends on the availability of information support services to resident and faculty at the point of patient care [17]. In conjunction with our EBM rotation we have also improved support for residents and faculty searching for evidence-based answers to clinical questions, through the development of our EBM-filtered information support website [15], access to a librarian information specialist (JH), and provision of PDAs to our interns who did not have their own.

We estimate that training 13 interns per year requires approximately 70 hours of librarian time and 200 hours of the faculty Director's time. In the event that the medical librarian Co-Director (JH) is not available, the faculty Director (DT) provides coverage for this portion of the rotation. Absence of the faculty Director for one or two days can usually be covered by schedule modifications and communication by telephone and e-mail. An extended absence of the faculty Director requires another faculty member assuming supervisory responsibility.

### Evaluation

We evaluated our EBM curriculum with respect to our 3 primary goals using pre- and post-rotation questionnaires (completed by each EBM intern) and by a survey of family practice residents and faculty.

The EBM intern questionnaires consisted of pre- and post-rotation self-assessments of confidence in EBM knowledge and skills (Goal 1). Self-confidence in EBM knowledge and skills was assessed by asking the intern to rate his or her level of comfort from 1 = very uncomfortable to 5 = very comfortable for (1) MEDLINE searching to answer a clinical question; (2) use of Web-based EBM resources to answer a clinical question; and (3) use of EBM principles to critically evaluate articles. At the end of the rotation interns were asked to identify the most useful and least useful aspects of the rotation and what could make the rotation better. In addition, residents completed and returned to the Residency Coordinator a standard evaluation of the rotation.

We evaluated the dissemination and reinforcement of EBM skills and knowledge to residents and faculty (Goal 2) and the application of EBM to patient care (Goal 3) by surveying residents (all of whom had previously completed the EBM rotation as interns) and faculty. These surveys were distributed and collected by a program assistant. Surveys were labeled with a code number for each resident and faculty and results were reported in aggregate to provide anonymity. The resident survey asked "How frequently have you continued to apply EBM concepts and tools from the rotation to answer clinical questions?" and "How frequently have EBM concepts and tools been reinforced via teaching by faculty or senior residents?" For both questions, response options were 1 = never, 2 = seldom (less than once per month on average); 3 = occasionally (1 to 3 times per month on average); 4 = often (1 or 2 times a week on average); and 5 = frequently (3 or more times per week on average). For faculty and residents who had had a clinical question answered by the EBM intern on the inpatient service were asked. "How often did the EBM answer provide useful information?" and "How often did the answer change your management of a patient?" For both questions, response options were 1 = less than 25% of the time; 2 = 25% to 75% of the time; and 3 = more than 75% of the time. Finally, all faculty and residents were asked if they believed that "the presence of the EBM rotation improves the quality of patient care within the family practice residency program."

## Results

During the period from July 1, 2001 to September 30, 2003, 30 EBM interns presented 30 journal clubs and generated 74 CATs in response to questions from the inpatient service. Journal club presentations and CATS are electronically archived and make accessible via the residency website [14]. Interns rated their experience during the rotation a median of 7 (superior) on a scale from 1 to 9. Written comments were also elicited and showed that one-on-one meetings with the EBM faculty (DH and JH) and exposure to web-based EBM resources were consistently valued. The only areas suggested for improvement were (1) to drop written material that repeated content of website tutorial (which we did) and (2) to have back-up EMB questions in the event that an appropriate question cannot be generated by the inpatient service (we have used this option only rarely). As shown in Table 1, residents' confidence in their level of EBM knowledge and skills significantly increased from prior to the rotation in all 3 areas assessed (Goal 1).

**Table 1:** Comparison of interns' confidence* in EBM skills pre- and post-rotation (n = 10)

	Mean Pre-Rotation Score	Mean Post-Rotation Score	Difference	P-value
Use of PubMed/Medline	4.11	4.78	+0.67	<.01
Use of other Web-based EBM resources	2.74	4.67	+1.93	<.001
Use of EBM tools and principles (critical appraisal)	2.58	4.44	+1.86	<.001
Mean confidence score	3.14	4.63	+1.39	<.001

* Assessed from 1 = very uncomfortable to 5 = very comfortable

Surveys to evaluate goals 2 and 3 were completed by 21 of 25 residents (response rate of 84%) and 12 of 13 faculty (response rate or 92%). As seen in Table 2, 86% of residents reported that they have continued to apply EBM concepts and tools learned in the rotation to clinical questions at least occasionally and 81% reported having had EBM concepts and tools reinforced occasionally or often by faculty or senior residents. Eleven of the 12 faculty (92%) agreed that the EBM curriculum had increased their use of EBM concepts and tools in teaching and 75% felt that the curriculum had increased their use of EBM concepts and tools in their own clinical practice.

**Table 2:** Residents' report of how often they applied EBM concepts and tools to patient care, and how often EBM concepts and tools are reinforced by senior residents and faculty (n = 21)

Item	Never	Seldom (< 1/month)	Occasionally (1 to 3 times/month)	Often (1 to 2 times/week)	Frequently (≥3 times/week)
	N (%)	N (%)	N (%)	N (%)	N (%)
Applied to patient care	0 (0)	3 (14)	7 (33)	8 (38)	3 (14)
Reinforced by senior residents and faculty	0 (0)	4 (19)	12 (57)	5 (24)	0 (0)

As shown in Table 3, most residents and inpatient attending faculty felt that the EBM answer to the inpatient clinical question provided useful information 25% to 75% of the time. Only 1 resident (and none of the faculty) reported the EBM answer provided useful information less than 25% of the time. The majority of the residents (65%) and faculty (83%) reported that the information led to a change in patient management 25% to 75% of the time, with the remainder reporting a change in management less than 25% of the time. All residents and 11 of the 12 faculty (92%) agreed that the EBM rotation had improved the quality of patient care within the residency program.

**Table 3:** Percent of time that the answer to EBM question provided useful information or led to a change in patient management on the Family Practice Inpatient Service

Item	Residents (n = 20*)			Faculty (n = 6**)		
	<25%	25% to 75%	>75%	<25%	25% to 75%	>75%

	N (%)	N (%)	N (%)	N (%)	N (%)	N (%)
Provided useful information	1 (5)	12 (60)	7 (35)	0 (0)	3 (50)	3 (50)
Led to a change management of the patient	7 (35)	13 (65)	0 (0)	1 (17)	5 (83)	0 (0)

* One Resident, who joined in the second year, had not been on the inpatient service during a time when an EBM intern was present.

** Answered by the 6 faculty members who attend on the inpatient service.

## Discussion

While the above findings support the acceptance and perceived utility of our EBM program, they do not provide a formal measure of its effectiveness. Changes in resident knowledge, skills and confident were measured over a short period of time. The frequency of reinforcement of EBM and the impact of EBM on clinical care was by resident and faculty report and may have been biased. No attempt was made to observe changes in physician behaviors or patient outcomes. There were no measures of EBM use prior to the introduction of the EBM rotation and no comparison group was available. It is therefore not possible to objectively determine to what degree current levels of awareness and utilization of EBM are the result of the rotation. It is also not possible to separate out the effects of the 2-week EBM rotation from the adjunct changes of establishing a medical information website or promoting the use of PDAs, except to the extent that the questions asked specifically about the 2-week EBM rotation.

Our block EBM rotation differs substantially from the approaches reported in previous studies [9,11,16] in that it utilizes a concentrated, individual experience that combines formal learning (via tutorials and a web-based course) with immediate application of EBM to answer an important clinical questions for individual patients – the target goal for EBM training. An advantage of our approach is the ability to tailor the curriculum to the background and needs of each intern and to provide interns with dedicated time during which they can rapidly acquire EBM knowledge and skills and apply them to "real time" clinical questions under the supervision of a faculty member and a librarian information specialist. We believe that our curriculum could be implemented by any faculty member with a working knowledge of EBM. The website tutorial [15] provides the core didactic portion for the rotation. Moreover, directing the rotation naturally increases the experience and expertise of the faculty member involved.

Interns reported greater confidence in their search skills after the two week rotation. Two randomized controlled trials and a controlled before-after study have demonstrated benefits of training in electronic search skills [17-19]. Interns also reported significantly greater confidence in their ability to apply EBM knowledge and principals in critical appraisal. This is consistent with previous studies that have found training improves critical appraisal skills for residents and practicing physicians [20].

One study that evaluated the impact of a 1-month pilot program to use EBM methods on an inpatient service, reported results roughly similar to ours [21]. Our curriculum also includes the archiving of EBM answers in a standardized format for future reference by residents and faculty. While our study did not include an evaluation of the usefulness of the archived EBM answers generated by the rotation, archiving EBM answers for web-based access has been shown to provide a useful resource for resident physicians in an internal medicine residency program [22].

Previous studies have found only small increases in residents' knowledge and skills from journal clubs alone, leading to the suggestion that journal clubs should be used as a component of EBM training rather then being 'stand alone' activities [6-8]. While we did not attempt to evaluate the effects of the journal club per se, including it as a component of our EBM curriculum is concordant with this suggestion.

Other investigators have reported educational interventions aimed at improving faculty knowledge and skills in medical informatics and EBM. One study reported increases in faculty self-rating of EBM skills following an intervention consisting of 2 half-day workshops and substantial amount of individual mentoring [23]. We found that faculty reported our EBM rotation has increased their use of EBM in their clinical practice, as well as their teaching of EBM; this was echoed by residents. While it is difficult to compare the two studies, our findings suggest that integrating EBM into the residency via resident training may improve faculty application of EBM to clinical care and in their teaching, and may be a cost-effective way to reinforce faculty EBM skills.

Our EBM curriculum, based on an individual 2-week EBM rotation in the first year, appears to be successful in increasing resident's EBM skills and confidence. In addition, resident and faculty both perceive EBM as being incorporated and reinforced beyond the rotation, and that the presence of EBM in the residency improves the quality of patient care. We hope that our experience provides a useful model for teaching and integrating EBM into a busy, resource-limited, family practice residency.

## Competing interests

The authors declare that they have no competing interests.

## Authors' contributions

All authors participated in the development of the curriculum. DT conceived of and conducted the evaluation. JH and PSS reviewed the survey instrument. JH and PSS reviewed and suggested changes to the paper. JH drafted the subsection describing the teaching the use of electronic. All authors have read and approved the final manuscript.

## Pre-publication history

The pre-publication history for this paper can be accessed here:


